# Therapeutic Touch in Exercise Videos: A Randomized Experiment of the Impact on the Evaluation of Therapists' Competence and Viewers' Self-Reliance

**DOI:** 10.3389/fspor.2019.00035

**Published:** 2019-09-25

**Authors:** Martina Bientzle, Janina Minje, Ulrike Cress, Joachim Kimmerle

**Affiliations:** ^1^Leibniz-Institut fuer Wissensmedien, Knowledge Media Research Center, Tübingen, Germany; ^2^PT Academy Tübingen, Tübingen, Germany; ^3^Department of Psychology, University of Tübingen, Tübingen, Germany

**Keywords:** therapeutic touch, exercise videos, self-reliance, evaluation, exercise instruction, self-efficacy

## Abstract

From a psychological health perspective, being physically touched is highly relevant throughout people's lives. Touch plays an important role in many contexts, such as in instructing movement exercises. Exercise videos have become a well-accepted format to support therapists in instructing movement exercises. In the study presented here we examined the impact of the use of therapeutic touch in exercise videos on people's evaluation of physiotherapists' competence and on their own self-reliance. In a between-group randomized experiment, 125 participants watched one of three videos that showed a physiotherapist who instructed a movement exercise to a patient. The physiotherapist touched the patient during the treatment (therapist-touch, TT), instructed the patient to use self-touch (ST), or provided only exercise instruction without physical touch (no-touch, NT). In the TT condition, the participants' perception was that the physiotherapist exhibited more professional competence. However, participants considered the movement exercise in this TT condition to have less potential for fostering their autonomy. Finally, participants in the ST condition had the biggest increase in perceived self-efficacy. The way of touching a patient in an exercise video influences the perception of the treatment. We conclude that therapeutic touch should be applied in exercise videos in a goal-oriented way: It seems appropriate to use ST if the aim is to strengthen viewers' self-reliance and to use TT to arouse trust in the competence of the therapist.

## Introduction

Videos have become a well-accepted format used by patients and medical experts to address health-related topics. Videos may provide even complex medical information in a cost-effective way (Sweat et al., 2001; Occa and Suggs, 2016; Eggeling et al., 2018; Grosser et al., 2018). They are therefore increasingly used for a wide range of purposes, such as giving instructions for movement exercises. As research has shown, video-based exercise instruction is more effective than static graphics for movement learning and home training (Weeks et al., 2002; Reo and Mercer, 2004). Moreover, video-based instruction can have a positive impact on viewers' self-confidence and motivation when carrying out movement exercises (Weeks et al., 2002). Even older people do not have any disadvantage when they receive additional instructions from videos (Schoo et al., 2005). Evidence suggests that videos support the (movement-) learning process and have mainly positive effects on compliance and motivation (Miller et al., 2009; Kingston et al., 2010). There is much research about convenient ways of designing instructional videos. Besides formal design principles of instructional videos (for an overview see Mayer, 2008) it is also important to take into account the personal characteristics of the models shown in the video, such as age and gender (Fleming and Ginis, 2004; Weeks et al., 2005; Hoogerheide et al., 2016). But instructing a movement exercise is comprised of more than formal aspects of the video and personal characteristics of the model. In many body-oriented therapies, like physiotherapy, different types of physical touch are an essential part of the treatment practice and the instruction of specific therapeutic movement exercises (Roger et al., 2002). Although the particular way of touching patients is assigned great importance in physiotherapy education and training (Roger et al., 2002; Nicholls and Holmes, 2012; Bjorbækmo and Mengshoel, 2016; Kelly et al., 2018), this aspect is not examined in research on instructional exercise videos. Little is known about how the use of therapeutic touch in exercise videos affects viewers' perceptions of the instructor and their own contribution to treatment success. So far nearly all studies that have been conducted in the context of body-oriented therapies used qualitative research methods, such as observations, interviews, or phenomenological approaches (Roger et al., 2002; Bjorbækmo and Mengshoel, 2016; Kelly et al., 2018). There is no data on potential causal effects of different types of therapeutic touch in instructional movement exercise videos. In the following sections, we provide a short overview of the role of therapeutic touch in general and in physiotherapy in particular. Subsequently, we derive hypotheses regarding the influence of therapeutic touch on the evaluation of physiotherapists' competence and viewers' self-reliance.

Being physically touched plays an important role throughout people's lives. Touch is central in early childhood development (Ferber et al., 2008). It has been shown that children in institutional care who often receive very little physical touch show retarded cognitive development (Field, 2010), and children of depressed mothers compensate for the lack of their mothers' touch with increased self-touch behavior (Herrera et al., 2004). Touch is also essential in social relationships: It may facilitate social interactions (Field, 2010), express emotions (Hertenstein et al., 2006, 2009; Thompson and Hampton, 2011), and lead to more complaisance (Crusco and Wetzel, 1984; Guéguen and Fischer-Lokou, 2003; Joule and Guéguen, 2007). To date there has been some empirical evidence that physical touch, and massages in particular, may lead to pain relief (Field, 1998; Kutner et al., 2008; Mancini et al., 2014; Alves et al., 2017), improve the mood of patients (Field, 1998; Kutner et al., 2008; Mancini et al., 2014; Alves et al., 2017), lower their heart rate (Field, 1998; Diego et al., 2004), reduce anxiety (Wilkinson et al., 2008; Post-White et al., 2009), and alleviate depressed moods (Krohn et al., 2011). Moreover, touch may have a direct influence on the healing process (Field, 1998; Weze et al., 2004) and emotional and physical well-being (Bush, 2001). However, the ways in which these effects occur are not well understood.

Even though the relevance of touch is not in doubt and also plays a role in many medical situations and for physical activities, there is no research about the role of touch in exercise instructions. It is known that beyond biomedical factors, therapeutic success also depends on attitudinal and motivational aspects, such as how patients perceive and evaluate their therapists (Ackerman and Hilsenroth, 2003; Lee and Lin, 2009) and how patients perceive their own self-reliance (Street, 2013; Slade et al., 2014; Carpenter et al., 2016). Touch and the perception of touch are also complex processes that are far from being entirely understood (Ackerley et al., 2014; Sathian, 2016). In addition, different types of therapeutic touch are used quite differently in various medical and exercise situations. It is apparent that the role of therapeutic touch is more complex than previously assumed. In the research presented here, we have examined an instructional video of a prototypical situation where therapeutic touch plays a prominent role, that is, in exercise instruction in physiotherapy.

A core task of physiotherapists is to teach movement exercises to improve body posture and the movement behavior of patients. In a given treatment situation, the exercise is selected according to physiotherapeutic findings (Bientzle et al., 2018). Touch is also used as a way of providing information (for different types of touch in physiotherapy see Roger et al., 2002) and as a “communicative tool” (Hiller et al., 2015). The concrete way in which physiotherapists touch their patients may have an impact on the patients' evaluation of therapists' competence and on their own self-reliance. Touch can be applied very differently for teaching movements and exercises: (1) Physiotherapists can omit physical touch altogether by demonstrating the movement exercise on their own body (no-touch, NT); (2) physiotherapists can touch patients to facilitate their exercise-learning processes (therapist-touch, TT); and (3) Physiotherapists can instruct patients to use self-touch (ST). This third way of movement instruction combines the tactile stimulus and the demonstration of a movement. Self-touch can be used by the patients themselves to facilitate the exercise-learning process. More senses of the patient are involved in the learning process with this method. As neurophysiological research has shown, touching one's own body requires additional processing of motoric information (Ackerley et al., 2012; Yang et al., 2012). Touching one's own body and being touched “engage similar afferent mechanoreceptors on the same skin site but may be processed differently” (Ackerley et al., 2012, p. 2). A main difference between self-touch and being touched by another person seems to be the amount of self-control of the touch along with the expectation of the impending sensory input associated with it. Perceived self-control and self-efficacy are highly relevant concepts when it comes to the extent to which people expect to be able to successfully carry out desired actions themselves on the basis of their own competencies. People with a high level of perceived self-efficacy believe that they can act independently even in difficult situations (Bandura, 1982, 1986). This, in turn, is extremely important when it comes burdensome duties, such as performing a physical exercise on a regular basis.

Whether and how therapists apply therapeutic touch in an exercise video may have an impact on how viewers evaluate the therapists' competence. Evaluation of competence would include people's perception of a therapist as being more or less friendly, caring, and empathetic (*social competence*), as well as their assessment of a therapist as being more or less skillful, thorough, and experienced (*professional competence*; Willson and McNamara, 1982; Bientzle et al., 2017). To achieve a sustainable effect, it is also important that viewers develop a certain level of self-reliance in terms of being able to conduct an exercise on their own (*autonomy* as a motivational prerequisite for maintaining an activity; Deci and Ryan, 1985, 2008) and in terms of having the impression that they can handle health-related problems by themselves (*self-efficacy*; Bandura, 1982, 1986; AbuSabha and Achterberg, 1997; Beierlein et al., 2013).

As outlined above, touch is relevant for facilitating social relationships. Therefore, we assumed that physiotherapists who touch their patients physically (TT) in an exercise video would be perceived as possessing more *social competence* than physiotherapists who do not touch their patients (ST and NT).

People usually also expect that physiotherapists will touch them physically during a therapeutic session. Accordingly, we hypothesized that physiotherapists who touch their patients (TT) in a video would be perceived as possessing more *professional competence* than physiotherapists who do not touch their patients (ST and NT).

In addition, we assumed that depending on the type of therapeutic touch, viewers would rate the movement exercise differently in terms of its potential to foster *autonomy*. If physiotherapists touch their patients during an exercise (TT), the viewers might get the impression that they are less able to conduct the exercise on their own than with physiotherapists who do not touch the patients (ST and NT).

Finally, we hypothesized that a video in which patients use self-touch during an exercise (ST) would give viewers a stronger impression that they can handle health-related problems on their own than settings in which patients are touched by the physiotherapist (TT) or settings without therapeutic touch (NT). We therefore hypothesized the largest increase in *self-efficacy* for the ST condition.

In sum, we stated the following hypotheses:

Perceived competence:

H1a: Social competence: TT > NT and ST

H1b: Professional competence: TT > NT and ST

Self-reliance:

H2a: Autonomy: TT < NT and ST

H2b: Increase in self-efficacy: ST > NT and TT.

## Method

### Participants

**Figure 1:**
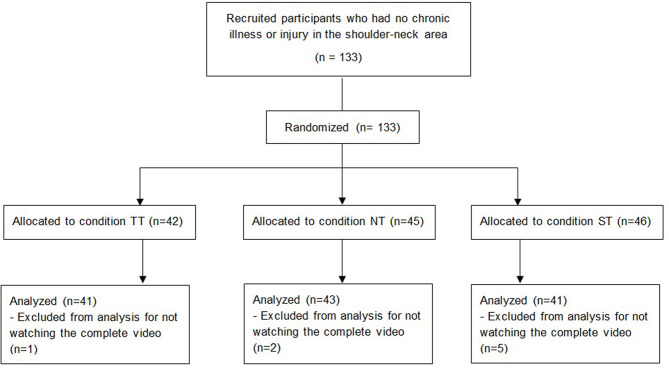
Flow diagram of study design.

We conducted a power analysis for ANOVAs in order to determine an adequate sample size for this study. Power analysis with α = 0.05, an intended power of 85%, and a medium to large effect size of f = 0.30 determined a target sample size of *n* = 126. Accordingly, we recruited 133 participants for this experiment. Eight participants had to be excluded as they had not watched the complete video (see Figure 1). The following analysis was based on the remaining 125 participants (age range: 19–69 years old, *M* = 26.47, *SD* = 10.06), of which 97 were women and 28 were men. This group of participants consisted predominantly of university students (*n* = 100; 80%). We randomly assigned 41 participants to the TT, 41 to the ST, and 43 to the NT conditions. The participants in the three conditions did not differ in terms of age and gender. All of the participants were recruited from the participant database of the Leibniz-Institut für Wissensmedien. They were invited via an e-mail that included a link to an online study. Only participants without any diseases or injuries in the shoulder and neck area were admitted to the study. Participation took about 20 min and was compensated with the opportunity to take part in a lottery, where participants could win one of ten vouchers worth 20 Euros each. All of the participants provided written informed consent. This research was performed in accordance with the Declaration of Helsinki and had full approval by the ethics committee of the Leibniz-Institut für Wissensmedien (approval number: LEK2016/016).

### Procedure

**Figure 2:**
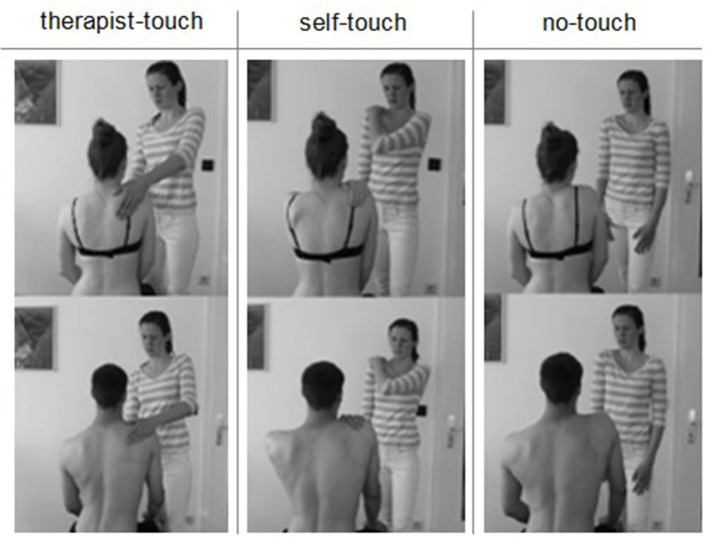
Study design and material (written informed consent was obtained from the individuals shown in this picture for the publication of this image). Female participants watched a video with a female patient (top row); male participants watched the identical physiotherapy treatment of a male patient (bottom row).

Initially, all participants were informed that they would participate in a study where they had to imagine themselves in a treatment situation in which a movement exercise was shown in a video. We chose a topic and title of the study that was relevant for many people (Ming et al., 2004): *Tense neck—what can I do?* All of the participants confirmed that they participated voluntarily, were informed about the purposes and the procedure of the study, and had no chronic illness or injury in the shoulder-neck area. Then we captured demographic data. After that, they filled in a pre-test questionnaire regarding their *self-efficacy* in matters of health. Subsequently, they were instructed to put themselves in the patient's place in a physiotherapy treatment. Female participants watched a video with a female patient; male participants watched the identical physiotherapy treatment of a male patient (see Figure 2). This procedure was used to facilitate their identification with the patient. Participants were randomly assigned to one of three conditions: They either watched a video that showed a physiotherapy treatment in which the physiotherapist touched the patient during the treatment (TT), instructed the patient to use self-touch (ST), or provided only exercise instruction without touch (NT). All videos were of comparable length (3.5–4 min). After watching the video, participants were asked if they had watched it to the end. Only those participants who confirmed this were considered for analysis. They were then asked to rate the *social competence* and *professional competence* of the physiotherapist in the video. They subsequently answered once again the *self-efficacy* questionnaire. Finally, participants evaluated the potential of the movement exercise for fostering patients' *autonomy*.

### Materials and Measures

#### Material

We scripted and filmed six videos with the same female physiotherapist. Three videos showed a female and three videos a male patient (see Figure 2). The male and female versions of the video were otherwise identical. All videos started with the welcoming of the patient. After being asked “How are you today?” the patient mentioned a tense neck. Then the physiotherapeutic intervention began. At first the physiotherapist asked the patient to take off the shirt and instructed the patient to sit upright. Next, the physiotherapist explained, based on anatomical information, a possible reason for the neck tension. The patient was asked to perceive the position of her/his own shoulder blades. Then a movement exercise for relaxation of the neck muscles was taught. The exercise was carried out three times in all of the conditions. The videos differed only in the usage of physical touch during the movement exercise. In the TT condition, the physiotherapist touched the patient at the shoulder and neck area during the exercise. The physiotherapist used a mixture of an assisted touch to aid the patient in the correct performance of the movement as well as a stroking of the tense muscle (see Roger et al., 2002). In the ST condition the physiotherapist instructed the patient to touch him-/herself in the same way as she demonstrated physically on herself. In the NT condition the physiotherapist provided only exercise instruction verbally without any physical contact.

After completing the exercise, the patient was asked to perceive the position of the shoulder blade again. In all conditions the patient in the video reported an improvement. After explaining the effect mechanism of the exercise, the physiotherapist invited the patient to repeat the exercise with the other shoulder. With this scene the video ended. The physiotherapist who was the instructor for the movement exercise, the structure of the video, and the information given in the six videos were identical.

#### Measures

We measured participants' perceived *social competence* with 10 adjective pairs that they rated on nine-point semantic differential scales, based on the measure by Willson and McNamara (1982). All items are shown in Table 1. The internal consistency was good (Cronbach's α = 0.92).

**Table 1:** Social competence scale.

Friendly	O	O	O	O	O	O	O	O	O	Unfriendly*
Discourteous	O	O	O	O	O	O	O	O	O	Courteously
Polite	O	O	O	O	O	O	O	O	O	Unpolite*
Unkind	O	O	O	O	O	O	O	O	O	Kind
Pleasant	O	O	O	O	O	O	O	O	O	Unpleasant*
Not likable	O	O	O	O	O	O	O	O	O	Likable
Considerate	O	O	O	O	O	O	O	O	O	Not considerate*
Insensitive	O	O	O	O	O	O	O	O	O	Sensitive
Sympathetic	O	O	O	O	O	O	O	O	O	Unsympathetic*
Unattractive	O	O	O	O	O	O	O	O	O	Attractive

Which adjectives describe the physiotherapist in the video correctly? Please tick the box that is closer to the word that the physiotherapist describes more accurately for you.

Asterisks (*): *indicate reversely coded items*.

To measure perceived *professional competence*, we used seven pairs of adjectives that participants rated on nine-point semantic differential scales, also following the Willson and McNamara (1982) measure. All items are shown in Table 2. The internal consistency was also good (Cronbach's α = 0.90).

**Table 2:** Professional competence scale.

Unskilled	O	O	O	O	O	O	O	O	O	Skillful
Experienced	O	O	O	O	O	O	O	O	O	Unexperienced*
Not thorough	O	O	O	O	O	O	O	O	O	Thorough
Accurate	O	O	O	O	O	O	O	O	O	Not accurate*
Incompetent	O	O	O	O	O	O	O	O	O	Competent
Educated	O	O	O	O	O	O	O	O	O	Not educated*
Confident	O	O	O	O	O	O	O	O	O	Not confident*

Which adjectives describe the physiotherapist in the video correctly? Please tick the box that is closer to the word that the physiotherapist describes more accurately for you.

Asterisks (*): *indicate reversely coded items*.

We measured the perceived potential of the exercise for fostering viewers' *autonomy* with three statements that participants had to rate according to how strongly they agreed with them, on a five-point Likert scale. The items are shown in Table 3. The internal consistency was acceptable (Cronbach's α = 0.69).

**Table 3:** Autonomy scale.

I can imagine that I can do the exercise shown here on my own now.
The exercise cannot be performed without a therapist*.
The exercise is appropriate to support the autonomy of the patient.

The asterisk (*): *indicates a reversely coded item*.

We measured health-related *self-efficacy* with three statements that participants had to rate according to how strongly they agreed with them, on a five-point Likert scale. The self-efficacy measure was based on Beierlein et al. (2013). All items are shown in Table 4. The internal consistency in the pretest (Cronbach's α = 0.86) and posttest (Cronbach's α = 0.88) was good. As we were interested in the increase of self-efficacy we calculated the difference between the pre- and the post-tests and used the resulting score for the following analysis.

**Table 4:** Health-related self-efficacy scale.

If I have health problems I can still rely on the abilities of my body.
I can cope with most health problems on my own strength.
Generally, I can deal well with health problems.


### Analysis

Data analysis was performed using IBM SPSS 20.0 for Windows (SPSS, 2011). The correlations among social competence, professional competence, autonomy, and self-efficacy are shown in Table 5. The means (*M*) and standard deviations (*SD*) are shown in Table 6. The level of significance was set at *p* < 0.05. Cohen's d is reported as effect size. To examine the influence of our manipulation on the evaluation of physiotherapists' competence and viewers' self-reliance, we calculated analysis of variance (ANOVA) with type of therapeutic touch as between-subject factor. To test the equality of variances we conducted Levene's test. In cases where the assumption of homogeneity of variances was violated, we calculated Welch tests (Kohr and Games, 1974). To test whether the sample came from a normally distributed population, we calculated Shapiro-Wilk tests for the dependent variables. In cases where the data were not normally distributed, we additionally tested our hypotheses with Mann-Whitney *U*-tests as a nonparametric test and reported the median as a measure of central tendency. Since simulation studies have shown that ANOVA is robust to violations of the normal distribution assumption (Schmider et al., 2010; Blanca et al., 2017), we also report contrast analyses to avoid multiple comparisons with less power (Furr, 2008) to test our specific hypotheses.

**Table 5:** Correlations among social competence, professional competence, autonomy, and health-related self-efficacy at the post-test.

	**Social competence**	**Professional competence**	**Autonomy**	**Health-related self-efficacy**
Social competence	–			
Professional competence	0.806 (*p* < 0.001)	–		
Autonomy	0.041 (*p* = 0.660)	0.082 (*p* = 0.378)	–	
Health-related self-efficacy	0.041 (*p* = 0.657)	0.008 (*p* = 0.931)	0.180 (*p* = 0.050)	–

**Table 6:** Means and standard deviations of the outcome variables in the three experimental conditions.

	**TT condition *M* (*SD*)**	**ST condition *M* (*SD*)**	**NT condition *M* (*SD*)**
Social competence	7.78 (1.18)	7.54 (1.04)	7.52 (1.18)
Professional competence	7.90 (1.15)	7.25 (1.34)	7.03 (1.52)
Autonomy	3.56 (1.14)	4.21 (0.70)	4.15 (0.67)
Increase in self-efficacy	0.12 (0.53)	0.30 (0.48)	0.06 (0.48)

## Results

**Figure 3:**
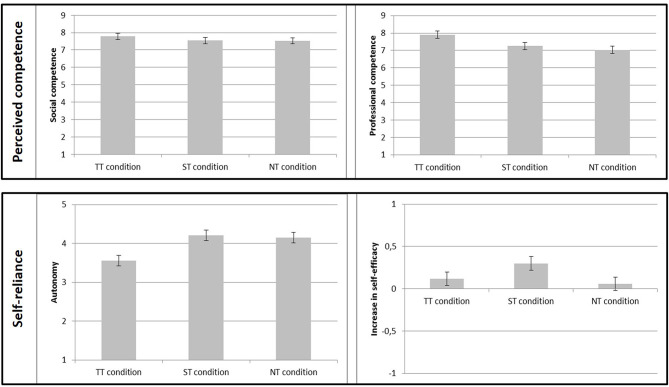
Impact of touch on perceived competence (social and professional competence) and self-reliance (autonomy and increase in self-efficacy). Standard errors are represented by error bars attached to each column.

An overview of the results for all outcome variables can be found in Figure 3. In H1a we had assumed that the TT condition would differ from the other conditions regarding perception of social competence. This hypothesis was not supported by the data [*F*_(2, 121)_ = 0.64, *p* = 0.531]. As social competence was not normally distributed as assessed by a Shapiro-Wilk test (*p* < 0.001), we additionally tested H1a with a Mann-Whitney *U* test and found the same pattern of results (TT vs. NT: *U* = 730.500, *p* = 0.236; TT vs. ST: *U* = 698.000, *p* = 0.248).

In H1b we had assumed that participants in the TT condition would rate the physiotherapist's professional competence to be higher than in the other conditions. The assumption of homogeneity of variances was violated (Levene's test, *p* = 0.029), so we calculated a Welch test. We found a significant main effect for touch type, Welch's [*F*_(2, 79.48)_ = 5.03, *p* = 0.009]. We conducted an orthogonal contrast that compared the TT condition to the ST and NT conditions (1|−0.5|−0.5). As expected, we found a statistically significant difference in perceived professional competence. Participants considered the physiotherapist in the TT condition to be more professionally competent than in the ST and the NT conditions, *p* = 0.001, *d* = 0.652. As professional competence was not normally distributed (*p* < 0.001), we additionally tested H1b with Mann-Whitney *U*-tests. We found the same pattern of results: Participants considered the physiotherapist in the TT condition (*median* = 8.14) to be more professionally competent than in the ST condition (*median* = 7.29, *U* = 574.500, *p* = 0.020) and more professionally competent than in the NT condition (*median* = 7.36, *U* = 555.500, *p* = 0.008).

The assumption of H2a was that participants in the TT condition would rate the movement exercise as being less able to foster their autonomy. Again, there was no homogeneity of variance (Levene's test, *p* < 0.001). We found a significant main effect, Welch's [*F*_(2, 74.74)_ = 5.05, *p* = 0.009.] We conducted an orthogonal contrast that compared the TT condition to the ST and NT conditions (1|−0.5|−0.5). As expected, we found a statistically significant difference in autonomy rating. Participants in the TT condition considered the movement exercise to have less potential for fostering autonomy than in the ST and the NT conditions, *p* = 0.002, *d* = −0.866. Since autonomy was not normally distributed (*p* < 0.001), we tested H2a also with Mann-Whitney *U* tests, which produced the same pattern of results: Participants considered the movement exercise in the TT condition (*median* = 3.67) to have less potential to foster autonomy than in the ST condition (*median* = 4.33, *U* = 526.000, *p* = 0.012) and in the NT condition (*median* = 4.17, *U* = 567.500, *p* = 0.024).

Finally, in H2b we had assumed that the increase in self-efficacy would be higher in the ST condition than in the other conditions. We found a tendency toward a main effect [*F*_(2, 117)_ = 2.55, *p* = 0.082]. We conducted an orthogonal contrast that compared the ST condition to the TT and NT conditions (−0.05|1|−0.5). The analysis showed that participants in the ST condition had a larger increase in self-efficacy than in the NT and the TT conditions, *p* = 0.015, *d* = 0.406. Increase in self-efficacy was not normally distributed (*p* < 0.001), so we additionally tested H2b with Mann-Whitney *U*-tests. Participants in the ST condition (*median* = 0.33) had a larger increase in self-efficacy than in the NT condition (*median* = 0.00, *U* = 567.000, *p* = 0.016), but not compared to participants in the TT condition (*median* = 0.00, *U* = 671.500, *p* = 0.189).

## Discussion

Being physically touched plays an important role throughout people's lives. Touch is relevant in many contexts, such as in instructing movement exercises. Exercise videos have become a well-accepted format to support therapists in instructing movement exercises. This experimental study examined the impact of therapeutic touch used in an instructional exercise video on the evaluation of therapists' competence and on viewers' self-reliance. Our results showed that the type of therapeutic touch elicited different effects: A therapist who touched her patient in a video was perceived as more *professionally competent* than a therapist who did not touch the patient. For people's self-reliance, in contrast, the physical touch of the therapist seems to be a hindrance: As hypothesized, participants in the TT condition rated the movement exercise as being less able to foster *autonomy*. This corresponds to the idea that an important difference between the ST condition and the other conditions is the level of self-control, which is relevant to an individual's expectation of successfully performing desired activities such as a movement exercise. This result could cautiously be interpreted as an indication that ST is beneficial in achieving a higher level of self-reliance.

The contrast analysis has shown that the increase in health-related *self-efficacy* was the highest in the ST condition. However, the non-parametric test only confirmed a significant difference between the ST and the NT conditions. The question remains whether the observation of touch itself had an impact on people's health-related self-efficacy and if perceived self-control can indeed explain the increase in self-efficacy. All in all, the effect on health-related self-efficacy should be considered with caution, since the ANOVA only showed a tendency toward a main effect.

We found no significant effect for *social competence*, even though professional and social competence are highly related constructs (see Table 5). A possible reason for this non-significant finding might be that observing physical touch is only a very subtle representation of therapeutic touch, and the power that was achieved in finding this small effect in our study was only *1-ß* = 0.17.

To our knowledge, this is the first study that has systematically investigated the impact of different types of therapeutic touch in instructional videos on the evaluation of competence and self-reliance in a randomized controlled experiment. There is generally very little research about the influence of different types of therapeutic touch in patient education and exercise instruction. In particular, ST is hardly ever taken into account in research, though it is commonly used in therapeutic settings. Therefore, this study is a first step toward a deeper understanding of the topic. Further studies that address therapeutic touch in different formats are required, such as instructional videos, pictures, or live-settings and in different contexts like the use of touch by different medical professions (e.g., physicians, nurses, or sports therapists).

The study presented here has some limitations. The scale for measuring autonomy was quite narrow in scope. The concept of autonomy also includes other aspects that could not be considered in the present study. In addition, the internal consistency of this scale was not particularly good. Nevertheless, autonomy and health-related self-efficacy were significantly correlated (see Table 5), which is an indication of the convergent validity of these scales.

Another limitation is that using touch in a video leads to a stronger focus of attention on the body region that is touched. This focus of attention could be confounded with other properties of touch. In future research, this aspect should be taken into account by at least highlighting the relevant body parts in the NT conditions.

## Conclusion

In conclusion, our study demonstrated that the way a physiotherapist touches a patient in a video can produce genuinely different effects on the evaluation of physiotherapists' professional competence, as well as on viewers' perceived autonomy and self-efficacy. Even though TT had an influence that led to a favorable evaluation of therapists' professionality, it had a negative impact on perceived autonomy. ST, in contrast, seems to be beneficial for viewers' health-related self-efficacy. Overall, the results suggest that the way of touching a patient in an exercise video can have an influence on the perception and success of the treatment. Particular means of therapeutic touch in instructional videos should therefore be applied consciously and in a target-oriented manner.

### Practice Implications

Therapeutic touch in instructional videos should be employed in accordance with the objective of the aim of the video. If a goal of the instructional video is to arouse trust in the competence of the therapist, the study findings suggest it would be appropriate to use TT to teach a movement exercise. However, when the main goal of therapy is to strengthen the self-reliance of the viewers, the study indicates that it seems to be more appropriate not to use TT and to instruct patients to use touch themselves during an exercise in order to strengthen viewers' self-efficacy. It is important that designers of instructional exercise videos learn about the impact of therapeutic touch on the assessment of the videos.

## Data Availability Statement

The datasets generated for this study are available on request to the corresponding author.

## Ethics Statement

The studies involving human participants were reviewed and approved by Ethics committee of the Leibniz-Institut für Wissensmedien. The patients/participants provided their written informed consent to participate in this study.

## Author Contributions

MB and JM were involved in the analysis and interpretation of data for the work. MB and JK drafted the work. JM and UC revised it critically for important intellectual content. All authors approved the final version to be published, agree to be accountable for all aspects of the work in ensuring that questions related to the accuracy or integrity of any part of the work are appropriately investigated and resolved and made substantial contributions to the conception and design of the work.

### Conflict of Interest

The authors declare that the research was conducted in the absence of any commercial or financial relationships that could be construed as a potential conflict of interest.
